# Phase II study of metronomic treatment with daily oral vinorelbine as first-line chemotherapy in patients with advanced/metastatic HR+/HER2− breast cancer resistant to endocrine therapy: VinoMetro—AGO-B-046

**DOI:** 10.1007/s00432-021-03599-2

**Published:** 2021-03-20

**Authors:** Slavomir Krajnak, Thomas Decker, Lukas Schollenberger, Christian Rosé, Christian Ruckes, Tanja Fehm, Christoph Thomssen, Nadia Harbeck, Marcus Schmidt

**Affiliations:** 1 Department of Gynaecology and Obstetrics, University Medical Centre, Mainz, Germany; 2 Haematology and Oncology Outpatient Clinic, Ravensburg, Germany; 3 Interdisciplinary Centre for Clinical Trials, University Medical Centre, Mainz, Germany; 4 Pierre-Fabre GmbH, Freiburg, Germany; 5 Department of Gynaecology and Obstetrics, University Medical Centre, Düsseldorf, Germany; 6 Department of Gynaecology, University Medical Centre, Halle (Saale), Germany; 7 Breast Centre, Department of Gynaecology and Obstetrics and CCC Munich LMU, University Hospital, LMU Munich, Munich, Germany

**Keywords:** Metronomic chemotherapy, Daily oral vinorelbine, Metastatic breast cancer, Clinical benefit rate

## Abstract

**Purpose:**

Metronomic chemotherapy (MCT) is an increasingly used treatment option in hormone receptor-positive (HR+)/human epidermal growth factor receptor 2-negative (HER2−) advanced/metastatic breast cancer (MBC) after failure of endocrine-based therapies.

**Methods:**

VinoMetro was a multicentre, open-label, single-arm, phase II study of metronomic oral vinorelbine (VRL; 30 mg/day) as a first-line chemotherapy (CT) in patients with HR+/HER2− MBC after endocrine failure. The primary endpoint was the clinical benefit rate (CBR) at 24 weeks.

**Results:**

Between January 2017 and April 2019, nine patients were enrolled. The CBR was 22.2% (90% confidence interval [CI] 4.1–55.0), *p* = 0.211. The median progression-free survival (PFS) was 12.0 weeks (95% CI 11.3–12.7). Grade 3–4 adverse events (AEs) occurred in 22.2% of patients. One patient died of febrile neutropenia.

**Conclusion:**

VinoMetro (AGO-B-046) was closed early after nine patients and occurrence of one grade 5 toxicity in agreement with the lead institutional review board (IRB). Metronomic dosing of oral VRL in HR+/HER2− MBC as first-line CT after failure of endocrine therapies showed only limited benefit in this population.

**Trial registration number and date of registration:**

ClinicalTrials.gov Identifier: NCT03007992; December 15, 2016.

## Introduction

With an incidence of 2.1 million and a mortality of 0.6 million cases per year, breast cancer (BC) represents a major disease burden worldwide (Bray et al. 2018). Hormone receptor-positive (HR+) and human epidermal growth factor receptor 2-negative (HER2−) disease represents the largest group of cancer subtypes (68%), with the majority of patients in this group showing a lower proliferation index (luminal A 44%; luminal B 24%) (Voduc et al. 2010). Advanced/metastatic breast cancer (MBC) is a treatable but still generally incurable disease (Cardoso et al. 2020; Thomssen et al. 2020). The goal of care in this situation is to reach a prolonged overall survival (OS) with adequate quality of life (QoL) and thus to establish a disease chronification using treatment options that provide an optimal therapeutic index (Harbeck and Gnant 2017). Single-agent chemotherapy (CT) is recommended as a preferred choice for patients with HR+/HER2−, non-life-threatening MBC after failure of endocrine treatment (Ditsch et al. 2020). Among available agents, vinorelbine (VRL) represents a standard treatment option in this situation, as it provides proven efficacy and a good safety profile (Fumoleau et al. 1993; Terenziani et al. 1996). Oral administration of VRL shows a level of efficacy comparable to the intravenous treatment, while providing a favourable safety profile and the additional advantages of an oral treatment (Aapro et al. 2019; Blancas et al. 2019; Freyer et al. 2003; Steger et al. 2018). Metronomic chemotherapy (MCT), describing the administration of low doses on a continuous and high administration frequency basis, has been shown to mediate good tumour control and maintain an excellent safety profile (Cazzaniga et al. 2019a; Krajnak et al. 2020; Liu et al. 2017; Orlando et al. 2020). This therapy option generally represents an approach of high interest in the treatment of solid tumours, as it offers the advantage of exposing the tumour to a significant amount of drug while improving safety for the patients (Cazzaniga et al. 2019b). It provides for fractionated, frequent, long-term administration of single doses of medication without pauses until disease progression or unacceptable toxicity (Gennari et al. 2011; Liu et al. 2017). MCT may have a complementary mechanism of action as compared to conventional CT, with an additional anti-angiogenic effect, thus counteracting tumour regrowth that may occur between conventional CT cycles (Kerbel and Shaked 2017; Natale and Bocci 2018). Moreover, MCT suppresses regulatory T cells (Tregs) and induces the maturation of dendritic cells, thereby leading to an anti-tumour immune response (Andre et al. 2017; Chen et al. 2017). The ease of oral VRL administration allows for flexible treatment schedules including a more frequent and metronomic dose application. Various schedules have been evaluated including a fractionated regimen (day 1, 3, 5) and daily intake (Adamo et al. 2019; Addeo et al. 2010; Guetz et al. 2017). In patients with non-small-cell lung cancer, the daily administration of VRL up to 40 mg per day was well-tolerated (Banna et al. 2018; Guetz et al. 2017). For these reasons, VinoMetro aimed to investigate a truly metronomic schedule with daily oral VRL in HR+/HER2− MBC patients following endocrine resistance, by assessing efficacy and safety of doses well below the maximum tolerated dose in advanced breast cancer patients with visceral metastases.

## Methods

### Study design

VinoMetro (ClinicalTrials.gov Identifier NCT03007992) was an investigator-initiated national, multicentre, open-label, single-arm phase II study sponsored by the University Medical Centre of the Johannes Gutenberg-University Mainz, Germany. It was initially planned to conduct the trial in 8 AGO-B (Arbeitsgemeinschaft Gynäkologische Onkologie–Breast) centres in Germany. As the study was stopped early upon request of the institutional review board (IRB) due to occurrence of one grade 5 toxicity, only two of these centres actually enrolled and treated patients between January 2017 and April 2019. The study was conducted in accordance with the 1987 Declaration of Helsinki and the Good Clinical Practice (ICH-GCP) guidelines. Approval of the protocol was obtained from the local ethics committee for each participating centre. Written informed consent was obtained from all patients prior to the performance of any trial specific procedure.

### Patients and treatment

Eligible patients were female, ≥ 18 years, with ECOG performance status ≤ 1 and estimated life expectancy ≥ 16 weeks. Further inclusion criteria were histologically confirmed BC and locally advanced or metastatic disease, previously untreated by palliative CT and not amenable to any curative treatment. Moreover, the included patients had to present with HR+ disease determined by ≥ 1% positive-stained cells for oestrogen receptor (ER) and/or progesterone receptor (PR) by immunohistochemistry (IHC) as well as HER2− disease (IHC 0–1 + or IHC 2 + , confirmed as FISH or CISH negative) in the primary tumour or a metastatic site. Only patients with relapse ≤ 12 months from end of adjuvant endocrine therapy or progression during/after the first line of endocrine therapy in the metastatic setting and/or being no longer a candidate for further endocrine therapy were included. Prior (neo-) adjuvant CT was allowed if the interval between end of CT and date of registration was > 12 months. Prior treatment with everolimus and/or cyclin-dependent kinase (CDK) 4/6 inhibitors as part of endocrine-based therapy was allowed. Presence of ≥ 1 measurable lesion as per RECIST 1.1 (Schwartz et al. 2016), which had not been previously irradiated as well as adequate bone marrow, hepatic and renal functions was required. The main exclusion criteria were prior vinca-alkaloids, aggressive disease-requiring combination CT and cerebral involvement. Patients with no recovery to ≤ grade 1 side effects (exception: alopecia) of any prior antineoplastic treatment, current peripheral neuropathy ≥ grade 2 and dysphagia or inability to swallow oral medication were also excluded.

Oral VRL (Navelbine® soft capsules) was administered at a daily dose of 30 mg (flat dose without any adaptation to body weight or body surface area) without breaks. One treatment cycle was defined as 28 days of therapy. Treatment was continued until disease progression, occurrence of unacceptable toxicity, patient’s refusal, or investigator’s decision to stop the treatment. Dose adjustments to 20 mg per day and dose delays were permitted in those patients who were unable to tolerate the dosing. Supportive care during the study was provided in accordance with established clinical standards and protocols. Blood tests including hematocrit, hemoglobin, red blood cell count (RBC), platelets, white blood count (WBC), differential (basophils, eosinophils, lymphocytes, monocytes, neutrophils) as well as clinical chemistry, including SGPT, SGOT, gamma-GT, alkaline phosphatase, total bilirubin, and creatinine, were performed weekly in the first two cycles and every two weeks afterwards.

### Study evaluation

The primary endpoint was the clinical benefit rate (CBR; complete response [CR] + partial response [PR]+ stable disease [SD]) at 24 weeks after start of metronomic treatment with daily oral VRL. Secondary objectives were to further assess the efficacy of metronomic VRL in terms of overall response rate (ORR; CR + PR), duration of disease control (DoDC), duration of stable disease (DoSD), progression-free survival (PFS), time-to-treatment-failure (TTF) and OS. Tumour measurements by computed tomography scan or magnetic resonance imaging were performed at screening (chest and abdomen/pelvis; within 28 days prior to the first intake of study medication) and repeated every 12 weeks (± 7 days) until end of study treatment. Whole-body bone scintigraphy and further potential imaging were performed according to clinical indication. Clinical response was determined using the revised RECIST guidelines version 1.1 (Schwartz et al. 2016). Assessment of safety and tolerability of metronomic VRL was conducted according to the National Cancer Institute Common Terminology Criteria for Adverse Events (NCI CTCAE) version 4.03.

### Statistical analysis

The minimum required level of efficacy (p0) was set to a CBR of 35% based on a prior trial investigating the standard-monotherapy with oral VRL in the first-line setting of patients with HR+/HER2− MBC (Freyer et al. 2003). An increase in CBR by 20% when using the metronomic treatment rated as being clinically relevant and accordingly the target CBR (p1) was set to 55%. Simon's two‐stage minimax design was used. The null hypothesis that the true response rate is 0.35 (p0) was tested against a one‐sided alternative. In the first stage, 21 patients were to be accrued. If there had been ≤ 8 patients with clinical benefit at 24 weeks in these 21 patients, the study would have been stopped. Otherwise, 18 additional patients would have been accrued for a total of 39. This design would yield a type I error rate of 0.05 and power of 0.80 when the true response rate was 0.55 (p1). Considering an anticipated dropout rate of approximately 15%, a total of 45 patients had to be accrued for this trial.

ORR and DCR were summarized as percentage rate and 95% confidence interval (CI). DoDC and DoSD were also analysed descriptively. PFS, TTF and OS were assessed using the Kaplan–Meier method.

## Results

### Patient characteristics

Between January 2017 and April 2019, 9 patients were recruited from two active cancer centres in Germany. Patient characteristics are listed in Table 1. The median age was 63.0 years (52.0–77.0). At the time of the first diagnosis (FD) of BC 2 (22.2%) and 7 (77.8%) patients had a T1 and T2 tumour, respectively. 3 (33.3%) patients were node-negative and 6 (66.7%) patients were node-positive (N1). None of the patients presented distant metastases at the time of FD. 5 (55.6%) and 4 (44.4%) tumours showed histological grade 2 and grade 3 cancer, respectively. 6 (66.7%) tumours were ER/PR-positive and 3 (33.3%) only ER-positive. 8 (88.9%) patients were postmenopausal. The median number of measurable metastatic lesions was 2.0 (1.0–3.0) with the liver being predominantly affected (9/17). The median size of these lesions was 50 mm and ranged from 15 to 71 mm. All patients presented with visceral metastases at the time of start of treatment. Local R0 surgical therapy as well as radiotherapy was performed in all patients within initial treatment (Table 2). 8 (88.9%) patients received adjuvant CT. Median number of prior lines of endocrine-based therapy was 3.0 (2.0–4.0) and the most common agents were tamoxifen (23.3%), letrozole (23.3%) and fulvestrant (20.0%).

**Table 1:** Patient characteristics

Characteristics		Patients (*n* = 9) (%)
Age (years)	Median	63.0
	Range	52.0–77.0
T_TNM	T1	2 (22.2%)
	T2	7 (77.8%)
	T3	0 (0.0%)
	T4	0 (0.0%)
N_TNM	N0	3 (33.3%)
	N1	2 (22.2%)
	N2	2 (22.2%)
	N3	2 (22.2%)
M_TNM	M0	9 (100.0%)
	M1	0 (0.0%)
Histological grade	G1	0 (0.0%)
	G2	5 (55.6%)
	G3	4 (44.4%)
HR status	ER-positive	9 (100.0%)
	ER-negative	0 (0.0%)
	PR-positive	6 (66.7%)
	PR-negative	3 (33.3%)
Menopausal status	Perimenopausal	1 (11.1%)
	Postmenopausal	8 (88.9%)
Measurable metastatic lesions	Median	2.0
	Range	1.0–3.0
Measurable metastatic lesions *n* (events) (*n* = 17)	Liver	9 (52.9%)
	Pleura	2 (11.8%)
	Cranium	1 (5.9%)
	Lymph nodes distant	3 (17.6%)
	Lymph nodes locoregional	1 (5.9%)
	Soft tissue	1 (5.9%)
Metastatic lesions *n* (patients)	Liver	9 (100%)
	Lung	1 (11.1%)
	Pleura	2 (22.2%)
	Peritoneum	1 (11.1%)
	Cranium	1 (11.1%)
	Bone	7 (77.8%)
	Lymph	3 (33.3%)
	Soft tissue	1 (11.1%)

*T* tumour size, *N* nodal status, *M* distant metastasis, *G* grade, *HR* hormone receptor, *ER* oestrogen receptor, *PR* progesterone receptor

**Table 2:** Prior therapy before study treatment

Therapy		Patients (*n* = 9) (%)
Surgical therapy	Yes	9 (100.0%)
	R0 surgery	9 (100.0%)
Adjuvant chemotherapy	Yes	8 (88.9%)
	No	1 (11.1%)
Adjuvant chemotherapy *n* (events) (*n* = 10)	EC	3 (30.0%)
	EC-paclitaxel	3 (30.0%)
	FEC-docetaxel	2 (20.0%)
	Other	2 (20.0%)
Endocrine-based therapy	Yes	9 (100%)
	No	0 (0.0%)
Lines of endocrine-based therapy	Median	3.0
	Range	2.0–4.0
Endocrine-based therapy *n* (events) (*n* = 30)	Tamoxifen	7 (23.3%)
	Letrozole	5 (16.7%)
	Letrozole/ CDK 4/6 inhibitor	2 (6.6%)
	Fulvestrant	5 (16.7%)
	Fulvestrant/CDK 4/6 inhibitor	1 (3.3%)
	Anastrozole	2 (6.7%)
	Exemestane	5 (16.7%)
	Leuprorelin/goserelin	3 (10.0%)
Radiotherapy	Yes	9 (100%)
	No	0 (0.0%)
Radiotherapy *n* (events) (*n* = 23)	Breast	6 (26.1%)
	Thorax wall	3 (13.0%)
	Lymphatic region	2 (8.7%)
	Axilla	2 (8.7%)
	Bone metastasis	10 (43.5%)

*EC* epirubicin/cyclophosphamide, *FEC* 5-fluorouracil/epirubicin/cyclophosphamide, *CDK* cyclin-dependent kinase

### Therapy response

**Fig. 1:**
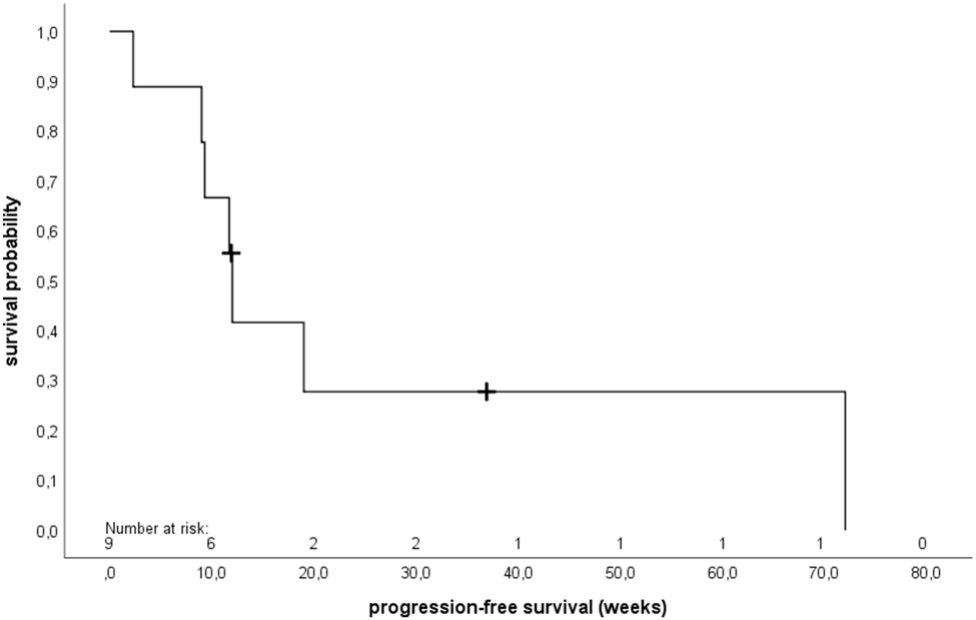
Kaplan–Meier analysis of progression-free survival (PFS) (*n* = 9). The median PFS was 12.0 weeks (95% confidence interval 11.3–12.7). + (censored)

**Fig. 2:**
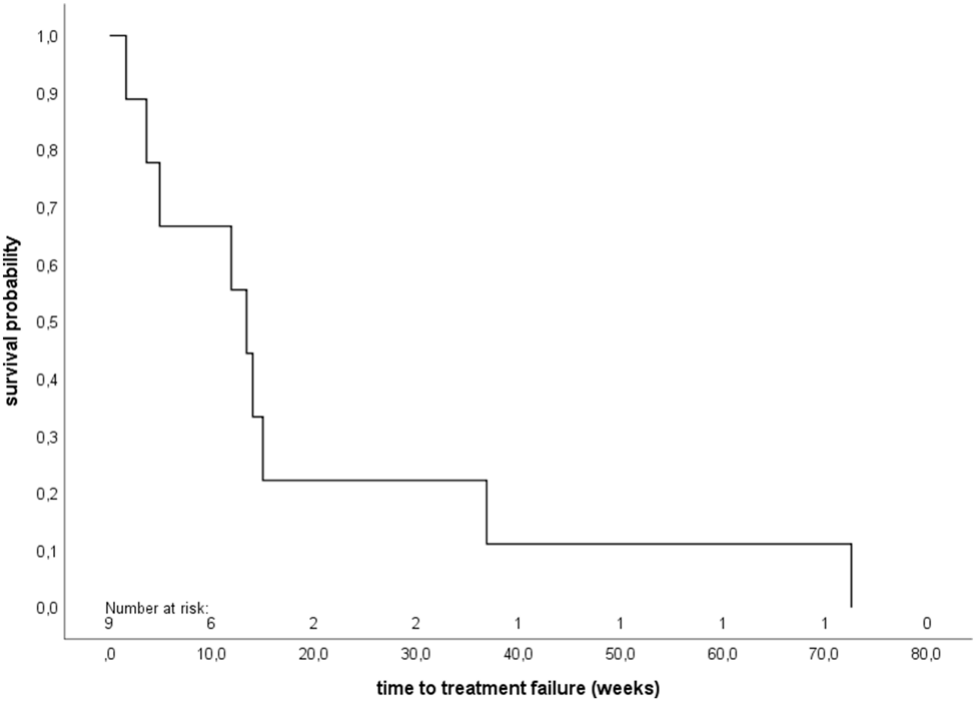
Kaplan–Meier analysis of time to treatment failure (TTF) (*n* = 9). The median TTF was 13.4 weeks (95% confidence interval 9.0–17.8)

The primary endpoint CBR at 24 weeks after start of metronomic treatment with daily oral VRL was 22.2% (90% CI 4.1–55.0), *p* = 0.211 (Table 3). There was no objective response achieved after 12 weeks of treatment. The results of DoDC and DoSD are identical because only SD could be achieved. Median DoDC/DoSD was 45.8 weeks (31.4–60.1). Median PFS was 12.0 weeks (95% CI 11.3–12.7) (Fig. 1), with a median TTF of 13.4 weeks (95% CI 9.0–17.8) (Fig. 2). Two patients who terminated the study early without opportunity for follow-up were censored in the calculation of PFS. Altogether three patients died, hence median OS could not be estimated.

**Table 3:** Therapy response

Clinical benefit rate at 24 weeks after start of vinorelbine		Patients (*n* = 9) (%)
	Yes	2 (22.2%)
	No	7 (78.8%)
	90% confidence interval	4.1–55.0
	*p* value (*p0* = 35%)	0.211
Duration of DoDC/DoSD (weeks)		Patients (*n* = 2)
	Mean (SD)	45.8 (20.3)
	Median	45.8
	Range	31.4–60.1
	Missing	7 (78.8%)
Adherence to therapy (%)	Median	91.0
	Range	49.0–100.0
Average daily dose (mg)	Median	27.3
	Range	14.7–30.0
Total amount of study medication taken (mg)	Median	2430.0
	Range	360.0–15, 120.0

Adherence to therapy = Percentage of patients who took the study medication according to the study protocol

*DoDC* duration of disease control, *DoSD* duration of stable disease, *SD* standard deviation

### Safety results

The median number of completed treatment cycles was 4.0 (0–18). One patient reached treatment cycle 18 and another one treatment cycle 10. All other patients discontinued the treatment at cycle 5 or earlier. The median adherence to therapy was 91% (49–100) (Table 3). The median average daily dose of VRL was 27.3 mg (14.7–30.0).

In total, 73 adverse events (AEs) were reported (8.1 per patient). 37 (50.7%) AEs were assessed as related to study treatment (4.1 per patient). The most frequent clinical AEs were nausea (55.6%), fatigue (44.4%) and diarrhoea (33.3%) (Table 4). Grade 3–4 AEs, including elevated liver enzymes, were documented in 2 (22.2%) patients. 1 (11.1%) grade 5 AE with a fatal outcome occurred as a consequence of neutropenic fever and pneumonia. The patient was 77 years old and had received tamoxifen and letrozole in combination with a CDK 4/6 inhibitor without (neo-) adjuvant CT prior to start of study treatment. The study medication was begun 4 weeks after prior therapy with normal blood values and was administered for only 12 days before the onset of pneumonia and febrile neutropenia. 4 days later, the patient died as a result of septic shock. After the occurrence of this grade 5 AE and taken into account the limited efficacy observed so far, it was decided in agreement with the lead IRB to terminate the study early without activating the other centres.

**Table 4:** Adverse events

Adverse event	Patients (*n* = 9) (%)			
	Overall	Grade 3	Grade 4	Grade 5
Neutropenia	1 (11.1%)	0	0	0
Anaemia	1 (11.1%)	0	0	0
Thrombocytopenia	0	0	0	0
Febrile infection	1 (11.1%)	0	0	1 (11.1%)
Fatigue	4 (44.4%)	0	0	0
Stomatitis	0	0	0	0
Nausea	5 (55.6%)	0	0	0
Vomiting	1 (11.1%)	0	0	0
Diarrhoea	3 (33.3%)	0	0	0
Constipation	0	0	0	0
Elevated liver enzymes	3 (33.3%)	2 (22.2%)	0	0

## Discussion

In VinoMetro, the observed CBR of metronomic VRL as a first-line CT in nine patients was 22.2%, well below the expected CBR. Moreover, after death of a patient in septic shock, the study was closed early in agreement with the IRB after careful risk / benefit analysis. To reduce potential publication bias, we decided to publish our results accordingly.

In the last years, therapy options for MBC increased steadily. Treatment choice should take several important factors into account. HR, HER2 status, germline BRCA status as well as PIK3CA mutation status in HR-positive and PD-L1 in triple-negative breast cancer (TNBC) should be assessed to allow for targeted therapies. Biological age, menopausal status, tumour burden, comorbidities and previous therapies with their toxicities are also crucial for decision-making (Cardoso et al. 2020; Thomssen et al. 2020). Nowadays, endocrine therapy combined with a CDK 4/6 inhibitor should be considered as a first choice in patients with HR+/HER2− MBC without life-threatening disease. In case of endocrine resistance, the guidelines recommend sequential single-agent CT as the preferred choice for MBC. Combination CT should be reserved for patients with rapid clinical progression, life-threatening visceral metastases, and/or the need for rapid symptom/disease control (Cardoso et al. 2020; Ditsch et al. 2020; Thomssen et al. 2020). Nevertheless, there still remains a high medical need for new therapy options in MBC that prolong the time between endocrine failure and CT, the latter being potentially associated with impaired QoL and more severe side effects. In this respect, the present phase II study evaluated the efficacy and safety of metronomic daily VRL. The primary endpoint CBR at 24 weeks after start of study treatment was 22.2%. There was no objective response achieved after 12 weeks of treatment. The median DoDC/DoSD was 45.8 weeks (31.4–60.1). The median PFS was 12.0 weeks (95% CI 11.3–12.7) and the median TTF was 13.4 weeks (95% CI 9.0–17.8).

These results in a small patient number showed only a limited efficacy of metronomic VRL compared to previous studies (Table 5). Addeo et al. treated 34 MBC patients with oral VRL 70 mg/m^2^ as first-line treatment, fractioned on days 1, 3 and 5, for 3 weeks on and 1 week off, every 4 weeks, for a maximum of 12 cycles. The median age was 75 years (70–84). The primary endpoint ORR was 38% (95% CI 28–48) and CBR at 12 weeks after start of treatment was 68% (95% CI 61–82). However, only 32% of patients suffered from visceral metastases compared to the present study where all patients showed visceral involvement (Addeo et al. 2010). In a study by De Iuliis et al., 32 MBC patients treated with oral VRL 30 mg every other day showed CBR of 50%. The median age was 76 years (69–83) and the patients were pre-treated with several CT lines (De Iuliis et al. 2015). In the VEX trial, Montagna et al. showed a significant activity and good tolerability of MCT in HR+MBC patients when VRL was administered in combination with cyclophosphamide (CTX) and capecitabine (CAPE). VRL 30 or 40 mg three times a week, CTX 50 mg once daily and CAPE 500 mg thrice daily received 43 patients as first-line CT and 65 patients as ≥ second-line CT. Visceral disease at the time of study inclusion was reported in 71% of patients. CBR for more than 6 months was 81% in the naive and 74% in the pre-treated group, the median time to progression (TTP) was 25.1 months (95% CI 14.2–39.1) and 11.2 months (95% CI 9.2–17.0), respectively (Montagna et al. 2017).

**Table 5:** Metronomic vinorelbine in patients with metastatic breast cancer

Study	Phase/clinical setting	Treatment	Number/age of patients (years)	Tumour	Results	
					Efficacy	Toxicity
Addeo et al. (2010)	II; first line	VRL (70 mg/m^2^) days 1, 3 and 5 (3 weeks on, 1 week off), q4w	34; median age (range) 74 (70–84)	ER+ or unknown status 62%	ORR 38% (95% CI, 28–48) mPFS 7.7 months (95% CI, 6.9–9.05) mOS 15.9 months (95% CI, 13.1–15.9)	Haematologic toxicity (grade 3/4) 24%; non-Haematologic toxicity (grade 3/4) 18%
De Iuliis et al. (2015)	II; several lines	VRL 30 mg one day on and one day off without interruptions	32; median age (range) 76 (69–83)	–	CBR 50%	No grade 3/4 toxicities
Cazzaniga et al. 2016, Victor-2 study	II; first line / ≥ second line	VRL 40 mg days 1,3 and 5, CAPE 500 mg thrice daily without interruptions	80 (35/45); median age (range) 66.3 (38.0–85.6) / 64.9 (44.0–82.7)	HR+ 65% Triple-negative 35%	CBR 45.7% (95% CI, 28.8–63.4) / 51.1% (95% CI, 35.8–66.3) ORR 35.5% (95% CI, 19.2–54.6) / 25.6% (95%CI, 13.5–41.2) mPFS 6.7 months (IQR, 4.7–11.3)/7.2 months (95% CI 2.8–11.5)	Haematologic toxicity (grade 3/4) 18%; non-haematologic toxicity (grade 3/4) 31%
Brems-Eskildsen et al. (2020), XeNa trial	II; first and second line	Arm A: VRL (60–80 mg/m^2^) day 1 and 8 + CAPE 2000 mg/m^2^ day 1–14 / Arm B: VRL 50 mg 3 times a week + CAPE 2000 mg/m^2^ day 1–14	118 ( 60 / 58); median age 60.8 / 60.9	ER+ 82% / 78%; all HER2−	CBR 46.8% / 51.7% mPFS 7.1 (95% CI, 3.9–10.3) / 6.3 (95% CI, 4.1–8.5) mOS 23.3 months (95% CI 20.2–26.4) / 22.3 months (95% CI, 14.3–30.3)	Haematologic toxicity (grade 3/4) 16% / 5%; non-haematologic toxicity (grade 3/4) 11% / 17%
Montagna et al. (2017), VEX trial	II; first line / ≥ second line	VRL 30–40 mg 3 times a week, CTX 50 mg daily, CAPE 500 mg thrice daily	108 (43 / 65); mean age (SD) 52.6 (10.1) / 55.2 (10.0)	HR+	CBR 81% / 74% mTTP 25.1 months (95% CI, 14.2–39.1) / 11.2 months (95% CI, 9.2–17.0)	Grade 3/4: 21% / 15%
Sanna et al. (2020)	Phase I; ≥ 1 prior line of therapy	VRL 20–40 mg 3 times per week, CTX 50 mg daily, bevacizumab 15 mg/kg q3w (HER2 + patients: + trastuzumab q3w)	15; median age (range) 61 (29–72)	ER + 80% HER2+ 33%	CBR 66.6% mPFS 6.9 months	Grade 3/4 toxicity 20%

*VRL* vinorelbine, *ER* + oestrogen receptor-positive, *ORR* overall response rate, *CI* confidence interval, *mPFS* median progression-free survival, *mOS* median overall survival, *CBR* clinical benefit rate, *CAPE* capecitabine, *HR* + hormone receptor-positive, *IQR* interquartile range, *HER2*− human epidermal growth factor receptor 2-negative, *CTX* cyclophosphamide, *SD* standard deviation, *mTTF* median time to treatment failure, *HER2*+ human epidermal growth factor receptor 2-positive

On the basis of favourable efficacy results with ORR of 4–31%, CBR of 49–56% and median PFS of 3.7–8.2 months, oral weekly VRL is considered as an active oral alternative to intravenous CT in the first-line CT treatment of MBC (Blancas et al. 2019; Freyer et al. 2003; Steger et al. 2018). It is noteworthy that these results are similar to the results of metronomic VRL regimens. In the VICTOR-2-study, MBC patients were treated with metronomic VRL 40 mg three times a week and CAPE 500 mg three times a day. The CBR was 48.8% (95% CI 37.4–60.2) and the median PFS was 6.7 months (95% CI 4.7–11.3) in the first-line treatment group and 7.2 months (95% CI 2.8–11.5) in the ≥ second-line treatment group (Cazzaniga et al. 2016). The efficacy of metronomic VRL 50 mg three times a week combined with standard CAPE treatment in HER2− MBC could be confirmed in the randomized phase II study XeNa. In the metronomic group with visceral involvement in 86% of patients, the CBR was 51.7% (95% CI 39.1–64.9) and the median PFS was 6.3 months (95% CI 4.1–8.5) without significant difference compared to the standard treatment (Brems-Eskildsen et al. 2020). Another retrospective study by Cazzaniga et al. which collected data from 584 h + /HER2− MBC patients treated with MCT showed an increased use of VRL-based regimens during the last years (2011: 16.8%—2016: 29.8%). 79.3% of patients received MCT as single agent. In the first-line setting, the highest ORR and DCR were observed for VRL-based regimens (single agent: 44% and 88%; combination: 36.7% and 82.4%, respectively). The median PFS was 7.2 months (95% CI 5.3–10.3) for VRL single agent and 9.5 months (95% CI 8.8–11.3) for VRL combinations. The median OS was 22.7 months (95% CI 13.0–43.5) for VRL single agent and 30.9 months (95% CI 26.2–34.7) for VRL in combination regimens (Cazzaniga et al. 2019b).

A meta-analysis of randomized trials including 2,269 MBC patients has shown that longer first-line CT duration is associated with marginally longer OS and a substantially longer PFS (Gennari et al. 2011). Thus, it is of high importance to find anticancer agents that could be administered for a long period without dose accumulation and accumulation of unacceptable side effects. In the present study 22.2% of patients presented grade 3–4 AE. More favourable results were reported in previous studies on MCT. The incidence of grade 3–4 events was 6–24%. The most frequent AEs grade 3–4 were anaemia and neutropenia (≤ 9%), elevated liver enzymes (5%) and gastrointestinal disorders (< 5%). Discontinuations due to AEs were observed up to 9% (Addeo et al. 2010, Cazzaniga et al. 2016, Cazzaniga et al. 2019b, De Iuliis et al. 2015, Montagna et al. 2017).

Most patients with incurable cancer prefer oral to intravenous therapy (Liu et al. 1997). A questionnaire assessing the perception of oral anticancer treatment could demonstrate a high acceptance of oral CT by most MBC patients. Oral administration helped the patients to feel less ill and to reduce the effort in coping with the disease (Catania et al. 2005). Moreover, metronomic VRL allows for easy daily or fractioned administration with the possibility of individual dose adjustment in case of toxicities and require less frequent hospital visits compared to standard intravenous CT (Gebbia and Puozzo 2005).

Taken together, there is evidence of increasing use of MCT, especially metronomic VRL, in MBC (Cazzaniga et al. 2019b; Sanna et al. 2020; Xu et al. 2020). However, randomized trials and phase III studies are lacking and there is currently not enough evidence about which regimen and which administration should be preferred (Cardoso et al. 2020; Cazzaniga et al. 2019a). The VinoMetro study did not confirm the results of previous studies of oral metronomic VRL, although daily oral administration has not been previously studied in MBC patients. One possible reason to explain these discrepancies could be the fact that all patients in VinoMetro had HR+ disease and visceral metastases. Another possible reason could be the daily administration of VRL as a single agent. Finally, given the small sample size, chance could be an additional explanation. Further insights with regard to metronomic VRL (fractionated regimen) are currently being generated in two randomized studies (TempoBreast: NCT03007992; TempoLung: EudraCT 2014-003859-61) comparing the metronomic with the conventional regimen in MBC and advanced non-small-cell lung cancer.

A weakness of our study is the limited sample size due to early study termination, thus the interpretation of the presented results is limited. A strength, however, is the prospective design evaluating for the first time the effectiveness and safety of daily administered low-dose metronomic VRL as first-line therapy in endocrine-resistant MBC with visceral metastases.

## Conclusions

This phase II study had to be closed early. The results in a small patient cohort showed only limited benefit of this treatment regimen in ER + /HER2− MBC patients with visceral metastases and progressive disease after endocrine-based therapy. The clinical relevance of the presented VRL administration in MBC should be evaluated in further trials.

## Data Availability

The datasets generated during the current study are available from the corresponding author on reasonable request.
